# Sociodemographic outcomes of childhood trauma experiences among young adults in the rural community of South Africa

**DOI:** 10.4102/hsag.v31i0.3271

**Published:** 2026-06-19

**Authors:** Bonolo Mmereki, Kebogile Mokwena, Mathildah M. Mokgatle

**Affiliations:** 1 Department of Public Health, Faculty of Health Sciences, Sefako Makgatho Health Science University, Pretoria, South Africa

**Keywords:** sociodemographic outcomes, childhood trauma, childhood traumatic events, mental health, childhood trauma questionnaire, Patient Health Questionnaire-9 (PHQ-9), depression symptoms, anxiety symptoms

## Abstract

**Background:**

Childhood trauma is a moving target, as its negative effects extend beyond the immediate mental health of individuals and continue to affect the victim years after it has occurred.

**Aim:**

The study aimed to determine the sociodemographic determinants of childhood traumatic events among young adults in the rural community.

**Setting:**

The study was conducted in South Africa.

**Methods:**

A quantitative, cross-sectional descriptive survey design was used to gather data among a sample of 380 participants in the rural community of the North West province. Data were collected using four sets of questionnaires to screen demographic determinants, childhood traumatic events and symptoms of depression and anxiety in the rural community of the North West.

**Results:**

With the probability set at *p* < 0.20, the findings showed that sociodemographic outcomes associated with childhood traumatic events are level of education, employment status, planning children, residence within the rural community, substance use, religious beliefs and depressive and anxiety symptoms.

**Conclusion:**

The findings of the study confirm that sociodemographic determinants are important factors in understanding the impact of childhood traumatic events.

**Contribution:**

This insight highlights the need for screening of childhood traumatic events. Depressive and anxiety symptoms should be prioritised in health facilities in South Africa.

## Introduction

Childhood trauma (CT) is a global psychosocial and public health problem that affects individuals younger than 18 years of age, with serious and everlasting effects on the current and future well-being of the victim and the broader society (Swedo et al. 2023). Childhood traumatic events are common in high-, middle- and low-income countries (Manyema & Richter 2019). Globally, more than 60% of people have experienced at least one childhood traumatic event in their lifetime, and one in six individuals have experienced more than four childhood traumatic events in their life (Verma & Agrawal 2021). The World Health Organization estimates that almost 5 million deaths are caused by trauma (Wyatt et al. 2017), and all these deaths affect some children.

Childhood traumatic events are experiences of abuse, neglect and household dysfunction varying in severity, duration and frequency (Maepa & Ntshalintshali 2020). This includes directly experiencing trauma, witnessing trauma or learning about trauma that happened to a close friend or relative. Among children, bullying, motor vehicle accidents, child maltreatment (neglect, physical, emotional and sexual abuse) and exposure to community and domestic violence are the common types of traumas that often result in distress later in adolescence and adulthood (Svecova et al. 2023).

South Africa has a relatively high prevalence of childhood traumatic events (Mall et al. 2020), with a reported 73.8% of the general population in South Africa having experienced at least one childhood traumatic event in their lifetime (Van Zyl et al. 2017). Children whose traumatic experiences have not received adequate attention in the form of specific interventions are at a higher risk of developing psychological conditions in adolescence and adulthood.

Peri-urban and rural communities in South Africa are especially characterised by psychosocial difficulties, physical violence, sexual violence and high prevalence of childhood traumatic events, because of high rates of violent crimes and makeshift houses within the community (Closson et al. 2016; Sharp et al. 2024). Children in these areas live in stressful environments characterised by racial discrimination and low socio-economic status, which increases their exposure to traumatic events in childhood (Liang et al. 2020).

Children exposed to trauma can experience delays in reaching their full potential, which can manifest as difficulties in learning at school, as well as problems with social, emotional and behavioural functioning (Frieze 2015).

Children who experience trauma are more likely to engage in risky sexual behaviours, such as engaging in alcohol and drug use, trading sex for money, having multiple sexual partners and experiencing intimate partner violence in adolescence (Calitz et al. 2014; Gibbs et al. 2018). Children may show symptoms such as replaying the trauma in play or behaviour, repeated memories of the event, distressing dreams, aggressive behaviour and negative thoughts about themselves and the future.

Although numerous studies have linked childhood traumatic events to several undesirable mental health outcomes in South Africa, there is a dearth of studies on interventions to combat the negative impacts of such events. Adequate social and family support is reported to Mitigate the negative effects of trauma among children and young adults (Stansfeld et al. 2017). However, there is a need in South Africa to screen young adults for CT to better understand its sociodemographic determinants and impact, as a significant proportion of mental health disorders emerge during this period.

## Research methods and design

### Study design

A quantitative cross-sectional descriptive survey method was employed for this study to determine the prevalence and the sociodemographic determinants of CT among young adults. This design was applied to the study in order to analyse data at a single point in time (youth attending primary health services at clinics) and surveys to gather numerical data from a large sample, allowing for statistical analysis to identify patterns and trends of CT in the rural community of North West.

### Population

The study was conducted among male and female young adults between the ages of 18 and 35 years who attended primary health services in Moretele health sub-district, South Africa.

### Study setting

The study was conducted in Moretele sub-district, which is a predominantly black residential area in the North West province. Five clinics, which included two 24h health centres, one 6day operating primary health clinic and two 5-day primary health clinics, were purposefully selected for the study. These clinics were specifically chosen because of the high influx of young adults attending the services provided, such as antenatal services, reproductive services, chronic services and youth zone services.

### Sample size and sampling techniques

A survey of clinic attendees was conducted and purposefully selected from five clinics in a predominantly black African rural residential area in the North West province, South Africa. The estimated headcount population from the five clinics was 10 000. Using the Raosoft sample size calculator (Raosoft Inc, Seattle, WA, United States), with a 5% margin of error, a 90% confidence level and a 50% response distribution, a minimum recommended sample size of 264 was calculated. The sample was buffered by 15% and increased to 303, but data were collected from 380 participants using purposive sampling, to intentionally select all young adults attending primary health services. Intentional oversampling was used to augment the minority group, which consisted of the participants who experienced CT in the general sample (Alkhawaldeh, Albalkhi & Naswhan 2023).

### Data collection process and tools

Data were collected by the researcher and research assistants. On the day of data collection, the data collectors were given private rooms within the clinics. They introduced themselves to the potential participants, verbally explained the purpose of the study and provided an opportunity for the participants to ask questions or seek clarifications. When the potential participants were satisfied with the information and were ready to participate in the study, they were requested to provide written informed consent, which was followed by the administration of the childhood trauma questionnaire (CTQ), the Patient Health Questionnaire-9 (PHQ-9) and the Generalised Anxiety Disorder-7 (GAD-7) questionnaires. At the end of the session, the researcher and assistants kept the questionnaires for later analysis.

The CTQ, developed by Pennebaker and Susman, was the primary data collection tool for the study. It contains six early traumatic experiences (death, divorce, child abuse, sexual abuse, illness and others). The total scoring of this CTQ is 84 using a 1–7-point scale to measure the trauma and the extent to which trauma was confided to. A score of 21 and above is classified as having CT.

The CTQ is a globally accepted self-reported questionnaire that provides retrospective screening for a history of childhood abuse and neglect (Wang et al. 2022). In Africa, the tool has been used in South Africa (Spies, Kidd & Seedat 2019), and its reliability and validity have been well documented (Jiang et al. 2018).

The PHQ-9 was the second main data collection tool for the study. It is a globally used self-reported administered questionnaire with nine items used to detect and quantify the severity of depressive symptoms (Maroufizadeh et al. 2019).

The GAD-7 was the third data collection tool for the study. It is a short, self-administered questionnaire that comprises seven questions and uses a 0–3-point scale (feeling anxious, inability to control worry, extreme worry about different things, trouble relaxing, restlessness, easily annoyed and feeling afraid) to identify the presence and severity of anxiety symptoms (Kigozi 2021).

### Data analysis

Data were captured on Microsoft Excel, cleaned up for incomplete questions, and then imported into Stata software (StataCorp 2015) for analysis. The demographic data were analysed descriptively, presented as tables and charts and shown as proportions and percentages. Descriptive statistics in the form of frequencies (*N*) and percentages (%) were used to describe the sample and to determine the prevalence of CT and its components. Fisher’s exact test was used to illustrate gender differences because the data were not normally distributed, and some of the cells had values less than 5.

Multivariate logistic regression was used to test for the association between CT, depression and anxiety.

### Ethical considerations

Ethical clearance to conduct this study was obtained from the Sefako Makgatho University Research Ethics Committee (SMUREC). The ethical clearance number is SMUREC/H/231/2023:PG. Furthermore, this study received permission from the provincial, district and sub-district levels of the Department of Health. Written informed consent was obtained from all participants. Confidentiality and respect were maintained throughout the administration of the questionnaires. No real names or any other identifiers were used to identify participants throughout the survey process.

## Results

### Description of sociodemographic determinants of childhood trauma

Table 1 reports demographic variables of participants by sex. The study included more female (90%) participants than male (10%) participants. In the current study, marriage rate was low among both females (89%) and males (97%), as both genders were not married. The majority of the participants had completed high school (62% females and 49% males). The unemployment rate was high among both female (86%) and male (62%) participants. Planning of children in the study was vital for female participants, as 58% planned their children prior to giving birth, compared to 68% of males who reported their children were not planned. Christianity was the most followed religious group; 74% of females and 51% of males were Christians.

**TABLE 1:** Sociodemographic characteristics of young adults by sex.

Variables	All *N* (%)		Female *n* (%)		Male *n* (%)		Fisher’s test
	*n*	%	*n*	%	*n*	%	
**Age (years)** 18–24 **25–35**	162 218	43 57	148 195	43 57	14 23	38 62	0.602 -
**Marital status** Not married **Married**	340 40	89 11	304 39	89 11	36 1	97 3	0.154 -
**Level of education** Primary school High school **Tertiary school**	129 231 20	34 61 5	114 213 16	33 62 5	15 18 4	40 49 11	0.121 - -
**Employment status** Unemployed **Employed**	321 59	84 15	298 45	86 13	23 14	62 37	0.001* -
**Recreational activities** None Sports **Community groups**	326 24 30	86 6 8	297 18 28	86 5 8	29 6 2	78 16 5	0.057 - -
**Planning of children** No **Yes**	169 211	44 56	144 199	42 58	25 12	68 32	0.005* -
**Number of children** < 2 **> 2**	304 76	80 20	270 73	79 21	34 3	92 8	0.001* -
**Social grants** No **Yes**	153 227	40 60	128 215	37 68	25 12	68 32	0.001* -
**Residence** **In** m**oretele** **Outside** m**oretele**	308 72	81 19	277 66	81 19	31 6	84 16	0.826 -
**Who raised you?** Nuclear family **Extended relatives**	304 76	80 20	278 65	81 19	26 11	70 30	0.131 -
**Substance use** None Alcohol **Smoking**	278 89 13	73 23 4	260 78 5	76 22 1	18 11 8	49 30 21	0.001* - -
**Religion** None Christian Muslim **African traditional**	92 275 2 11	24 72 1 3	78 256 2 7	23 74 1 2	14 19 0 4	38 51 0 11	0.002* - - -
**CT before 18 years** Yes **No**	36 344	9 91	31 312	9 91	5 32	14 86	0.377 -
**Symptoms of depression** Yes **No**	45 335	12 88	41 302	11 80	4 33	1 8	1.000 -
**Symptoms of anxiety** Yes **No**	31 349	8 92	29 314	7 83	2 35	1 9	0.754 -

Fisher’s test,

* , *p* < 0.005.

CT, childhood trauma.

### The types of childhood trauma experienced prior to the age of 18 years (*N* = 36)

Table 2 reports the types of CT that participants experienced; 36 participants had a score of 21 and above on the CTQ and 30% experienced death of a loved one or parents, 22% experienced divorce or separation of parents, 17% experienced sexual assault, 14% experienced child abuse, 11% experienced a major illness and 6% experienced a major Upheaval.

**TABLE 2:** Types of childhood trauma experienced prior to the age of 18 years (*N* = 36).

Category	Frequency (*N*)	Percentage (%)
Death of parents or loved ones	11	30
Divorce or separation of parents	8	22
Sexual abuse	6	17
Child abuse	5	14
Extreme illness	4	11
Major life-changing trauma	2	6

**Total**	**36**	**100**

*Source*: Childhood trauma questionnaire by Pennebaker, J.W. & Susman, J.R., 1988, ‘Disclosure of traumas and psychosomatic processes’, *Social Science and Medicine* 26(3), 327–332. https://doi.org/10.1016/0277-9536988)90397-8

### Comparison of demographics of the adolescents by childhood trauma (*N* = 380)

Table 3 reports a comparison of variables with CT. Probability was set at *p* < 0.20. Only significant variables were included in the table: level of education, employment status, participants who had their children planned, those who resided within the rural community, substance use and religion. Depression and anxiety were the two mental health conditions that were associated with CT in this study.

**TABLE 3:** Comparison of demographics by childhood trauma (*N* = 380).

Variables	All *n* (%)		CTQ No *n* (%)		CTQ Yes *n* (%)		*p*-value
	*n*	%	*n*	%	*n*	%	
**Level of education** Primary school High school **Tertiary school**	129 231 20	34 61 5	121 207 16	35 60 5	8 24 4	22 67 11	0.110* - -
**Employment status** Unemployed **Employed**	321 59	84 15	293 51	85 15	28 8	78 22	0.244* -
**Planning of children** No **Yes**	169 211	44 56	146 198	42 58	23 13	64 36	0.014* -
**Residence** **In moretele** **Outside moretele**	308 72	81 19	276 68	80 20	32 4	89 11	0.207*
**Substance use** None Alcohol **Smoking**	278 89 13	73 23 4	256 78 10	74 23 3	22 11 3	61 31 8	0.108* - -
**Religion** None Christian Zion, Apostolic & Muslim **African traditional**	92 227 50 11	2 60 13 3	90 197 49 8	26 58 14 2	2 30 1 3	6 83 2 8	0.001* - - -
**Depression** No **Yes**	335 45	88 12	312 32	91 9	23 13	64 36	0.001* -
**Anxiety** No **Yes**	349 31	92 8	323 21	94 6	26 31	72 28	0.001* -

CTQ, childhood trauma questionnaire.

Probability set at

* , *p* < 0.20.

### Logistic regression model

Table 4 presents the results of the logistic regression analysis. Bivariate analysis was used to analyse sociodemographic outcomes associated with childhood, and five demographics had a *p*-value of less than 0.05. These were the level of education, having planned children, substance use, symptoms of depression and anxiety symptoms. An adjusted multivariate model was built to confirm the model’s validity with associated variables. Results showed that the adjusted odds ratio (AOR) values and confidence interval (CI) ranges were as follows: level of education: AOR = 1.35; 95% CI: 0.68–2.69, planning of children: AOR = 0.045; 95% CI: 0.21–0.97, young adults who used substances: AOR = 1.50; 95% CI: 0.81–2.75, participants who had symptoms of depression: AOR = 3.08; 95% CI: 1.01–9.43 and anxiety symptoms: AOR = 2.42; 95% CI: 0.70–8.26.

**TABLE 4:** Logistic regression analysis of sociodemographic factors related to experiences of childhood trauma among young adults.

CT	Unadjusted odds ratios			Adjusted odds ratios		
	OR	*p*-value	95% CI	AOR	*p*-value	95% CI
Level of education	1.886996	0.048	1.00–3.54	1.35868	0.379	0.68–2.69
Employment	1.641459	0.248	0.70–3.80	1.816841	0.214	0.70–4.65
Planning of children	0.4167765	0.016	0.20–0.85	0.4545207	0.042	0.21–0.97
Residence	0.5073536	0.215	0.17–1.48	0.7818156	0.671	0.25–2.43
Substance use	1.764383	0.047	1.00–3.08	1.500139	0.192	0.81–2.75
Religion	1.505634	0.085	1.16–2.39	1.93751	0.011	1.16–3.22
Depression	5.51087	0.001	2.54–11.91	3.089114	0.048	1.01–9.43
Anxiety	5.915751	0.001	2.52–13.87	2.421223	0.158	0.70–8.26

CT, childhood trauma; OR, odds ratio; CI, confidence interval; AOR, adjusted odds ratio.

* , *p* < 0.05.

## Discussion

Social determinants of health are circumstances where people live, play, work and learn that affect health and outcomes in life (Johnson-Motoyama et al. 2022). In the current study social determinants of health that were associated with childhood trauma are; education, employment status, participants who planned their children, residence, substance use, religion, depression and anxiety.

Understanding these sociodemographic factors is imperative for the prevention of adverse events of CT in late adulthood.

Children and adolescents who experience trauma have difficulty in learning; trauma remains stressful for individuals, making it difficult for them to concentrate, causing an inability to concentrate and learn at school, resulting in poor academic performance (Singh 2024). In our study, there was a significant association between CT and the level of education, similar to a few other studies (Fung, Chung & Ross 2020; Lecy & Oesteen 2022). The integration and collaboration of trauma-based interventions is essential in schools to identify learners at risk of CT, which may diminish school dropouts and poor academic outcomes.

Our results showed that individuals who were unemployed were more likely to have CT. This is in line with other research results where CT increased the risk of unemployment in later adulthood (De Vries et al. 2023; Petersen et al. 2022). The stress sensitisation theory states that having experienced adverse childhood events reduces a person’s ability to cope during stressful times, which may increase the risk of lower educational achievement because of stress, resulting in decreased economic productivity and unemployment during adulthood (Petersen et al. 2022).

Planning of children prior to their birth was associated with CT, similar to a study done where planning and having one child or two children was greatly associated with CT (Chamberlain et al. 2019; Tong et al. 2022). Prior to having children, adequate support from family, partner and friends may foster a positive approach and diminish the stress and worry associated with the trauma (Mathijssen, Dirks & Van Bakel 2024). Most parents with a history of CT are able to provide caring environments for their children; this is because of being overprotective and choosing to parent differently from their previous experiences (Chamberlain et al. 2019).

Contrary to our study, a systematic review showed that mothers with a history of CT found themselves with unintended first pregnancies. This may be the result of a lack of social support, emotional regulation and modelling of healthy relationships to rely on.

The study was conducted in a rural community; staying and growing up in a rural community may predispose individuals and children with many adversities such as poverty, lack of access to resources, parental unemployment and lack of access to healthcare services that may impact their development and growth and increase the likelihood of children witnessing interpersonal violence, household accidents, etc. (Frost et al. 2024). Our study showed associations between CT and residence, which is in line with other studies that revealed that growing up in a rural setting was associated with CT (Frost et al. 2024; Hatcher et al. 2019).

Research reports that adolescents and young adults with CT engage in risky behaviours such as alcohol and drug use more than those without CT (Cheng et al. 2021; Meulewaeter, De Pauw & Vanderplasschen 2019; Rogers et al. 2023). This is consistent with our results as young adults with a history of childhood traumawere more likely to use substances than those without childhood trauma. It is found that people who experience trauma use substances as self-medication to reduce the pain of the trauma (Meulewaeter et al. 2019). The use of substances among adolescents and young adults reduces the ability to function socially, psychologically and physically over time. Social support in school and homes was found to diminish substance use among these groups (Cheng et al. 2021).

Religion and spiritual beliefs play an important role in how people react to CT. Individuals with a strong spiritual foundation have a sense of peace, better cognitive processes and a positive and strong behaviour, which foster identity formation and ethical development, thereby mitigating the effects of trauma later in adulthood (Nowicki et al. 2023; Tatala, Wojtasinski & Rynio 2024). The results of our research confirm this finding, as many young adults in the study were part of religious groups. The religious worship and prayers offered in Christianity and other religions, such as Islam, have been found to help ease the effects of trauma in survivors later in adulthood, yielding to a more positive mental health psychopathology and lowered anxiety (Kosarkova et al. 2020; Upenieks et al. 2024).

However, other studies documented negative religious emotions where people became angry with God for allowing CT to occur, seeing the trauma experience as punishment, feeling of abandonment by God and feeling that their sense of protection from God is threatened (Wolkinson & Weinberg 2023). Religion, therefore, plays an important role in reaction to trauma, and a high and positive religious foundation prior to and during CT has been linked to better mental health outcomes (Nowicki et al. 2023).

Experiencing internal trauma during childhood may affect brain functioning and lead to chronic and persistent negative emotions, which may manifest as depression if not given proper medical attention (Wang et al. 2023).

Depression and anxiety symptoms were associated with CT in the current study. Research studies show that chronic depression is linked to having experienced at least one type of childhood traumatic event, and a significant correlation between anxiety and CT has been noted (Kascakova et al. 2020; Li, Tu & Jiang 2022; McLaughlin et al. 2020).

The onset of poor mental health outcomes because of childhood traumatic events may result from a combination of poor brain processes, psychological factors, genetic predispositions and the environmental impact in later adulthood (Kuzminskaitse et al. 2021). Therefore, mental health assessment and screening for children and adolescents in health facilities is imperative to detect the onset of depression and anxiety symptoms before they develop into chronic conditions.

### Recommendations

The administration and screening of the CTQ, PHQ-9 and GAD-7 forms in all primary health facilities will ensure early detection and management of CT, depression and anxiety among children, young people and other attendees. This will help provide proper interventions, such as counselling therapy, pharmacological therapy and referrals to mental health institutions, to all victims with signs of CT in order to prevent mental health disorders later in adolescence and adulthood.

Public health strategies focusing on child, adolescent and adult health should be given priority in all health facilities in South Africa.

### Limitations of the study

The study was contextual in the North West in South Africa (Moretele sub-district); thus it may not represent the whole country. Childhood trauma is a sensitive aspect, and CTQ was used to recruit participants with CT from healthcare facilities only; therefore, the sample may not represent the whole population affected by CT.

## Conclusion

In conclusion, CT is one of the most significant social and health burdens in South Africa as it affects many children before the age of 18 years, as well as adolescents and adults. Sociodemographic factors are associated with CT, and they are essential for identifying and understanding the impact and types of CT affecting individuals. The effects of CT may result in mental health psychopathology, such as depression and anxiety, later in life if proper interventions are not provided after the traumatic event.

## References

[CIT0001] Alkhawaldeh, I.M., Albalkhi, I. & Naswhan, A.J., 2023, ‘Challenges and limitations of synthetic minority oversampling techniques in machine learning’, *World Journal of Methodology* 13(5), 373–378. 10.5662/wjm.v13.i5.373 38229946 PMC10789107

[CIT0002] Calitz, F.J.W., De Jongh, N.J., Horn, A., Nel, M.L. & Joubert, G., 2014, ‘Children and young adults treated for post-traumatic stress disorder at the Free State Psychiatric Complex’, *South African Journal of Psychiatry* 20(1), 15–20. 10.4102/sajpsychiatry.v20i1.441

[CIT0003] Chamberlain, C., Gee, G., Harfield, S., Campbell, S., Brennan, S., Clark, Y. et al., 2019, ‘Parenting after a history of childhood maltreatment: A scoping review and map of evidence in the perinatal period’, *PLoS One* 14(3), 1–41. 10.1371/journal.pone.0213460 PMC6415835 30865679

[CIT0004] Cheng, S., Wen, Y., Liu, L., Cheng, B., Liang, C., Ye, J. et al., 2021, ‘Traumatic events during childhood and its risk to substance use in adulthood: An observational and genome-wide by environment interaction study in UK Biobank’, *Translational Psychiatry* 11(431), 1–6. 10.1038/s41398-021-01557-7 34417442 PMC8379203

[CIT0005] Closson, K., Dietrich, J.J., Nkala, B., Musuku, A., Cui, Z., Chia, Z. et al., 2016, ‘Prevalence, type and correlates of trauma exposure among adolescent men and women in Soweto, South Africa: Implications for HIV prevention’, *BMC Public Health* 16(1191), 1–15. 10.1186/s12889-016-3832-0 27884181 PMC5123224

[CIT0006] De Vries, T.R., Arends, I., Oldehinkel, A.J. & Bultmann, U., 2023, ‘Associations between type of childhood adversities and labour market participation and employment conditions in young adults’, *Journal of Epidemiol Community Health* 77(4), 230–236. 10.1136/jech-2022-219574 PMC10086506 36805940

[CIT0007] Frieze, S., 2015, ‘How trauma affects student learning and behaviour’, *Journal of Graduate Studies in Education* 7(2), 27–34.

[CIT0008] Frost, A., Collins, A., Chung, E.O., Carias, M.S.E., Hagaman, A., Gupta, S. et al., 2024, ‘Trauma exposure among young children in rural Pakistan: Associations with gender, mental health and cognitive skills’, *BioMed Central Psychology* 12(454), 1–10. 10.1186/s40359-024-01944-x PMC11346172 39183356

[CIT0009] Fung, H.W., Chung, H.M. & Ross, C.A., 2020, ‘Demographic and mental health correlates of childhood emotional abuse in a Hong Kong sample’, *Child Abuse and Neglect* 99(104288), 1–10. 10.1016/j.chiabu.2019.104288 31821980

[CIT0010] Gibbs, A., Dunkle, K., Washington, L., Willian, S., Shai, N. & Jewkes, R., 2018, ‘Childhood traumas as a risk factor for HIV-risk behaviours amongst young women and men living in urban informal settlements in South Africa: A cross-sectional study’, *PLoS One* 13(4), 1–12. 10.1371/journal.pone.0195369 PMC5889178 29624612

[CIT0011] Hatcher, A.M., Gibbs, A., Jewkes, R., McBride, R.S., Peacock, D. & Christofides, N., 2019, ‘Effects of childhood poverty and trauma on adult depressive symptoms among young men in Peri-urban South African settlements’, *Journal of Adolescent Health* 64, 79–85. 10.1016/j.jadohealth.2018.07.026 30327276

[CIT0012] Jiang, W.J., Zhong, B.L., Liu, L.Z., Zhou, Y.J., Hu, X.H. & Li, Y., 2018, ‘Reliability and validity of the Chinese version of the childhood trauma questionnaire-short form for inpatients with schizophrenia’, *PLoS One* 13(12), 1–9. 10.1371/journal.pone.0208779 PMC6292582 30543649

[CIT0013] Johnson-Motoyama, M., Moon, D., Rolock, N., Crampton, D., Nichols, C.B., Haran, H. et al., 2022, ‘Social determinants of health and child maltreatment prevention: The family success network pilot’, *International Journal of Environmental Research and Public Health* 19(22), 1–8. 10.3390/ijerph192215386 PMC9692748 36430105

[CIT0014] Kascakova, N., Furstova, J., Hastova, J., Madarasova Geckova, A. & Tavel, P., 2020, ‘The unholy trinity: Childhood trauma, adulthood anxiety and long-term pain’, *International Journal of Environmental Research and Public Health* 17(2), 1–14. 10.3390/ijerph17020414 PMC7013389 31936285

[CIT0015] Kigozi, G., 2021, ‘Construct validity and reliability of the generalised anxiety disorder-7 scale in a sample of tuberculosis patients in the Free State Province’, *South Africa, Southern African Journal of Infectious Diseases* 36(1), 1–6. 10.4102/sajid.v36i1.298 PMC8424773 34522696

[CIT0016] Kosarkova, A., Malinakova, K., Koncalova, Z., Tavel, P. & Van Dijk, J.P., 2020, ‘Childhood trauma is associated with the spirituality of non-religious respondents’, *International Journal of Environmental Research and Public Health* 17, 1–11. 10.3390/ijerph17041268 PMC7068247 32079153

[CIT0017] Kuzminskaitse, E., Penninx, B.W.J.H., Van Harmelen, A.L., Elzinga, B.M., Hovens, J.G.F.M. & Vinkers, C.H., 2021, ‘Childhood trauma in adult depressive and anxiety disorders: An integrated review on psychological and biological mechanisms in the NESDA cohort’, *Journal of Affective Disorders* 283, 179–191. 10.1016/j.jad.2021.01.054 33561798

[CIT0018] Lecy, N. & Oesteen, P., 2022, ‘The effects of childhood trauma on college completion’, *Research in Higher Education* 63(6), 1058–1072. 10.1007/s11162-022-09677-9

[CIT0019] Li, X., Tu, L. & Jiang, X., 2022, ‘Childhood maltreatment affects depression and anxiety, the mediating role of benign envy and malicious envy’, *Frontiers in Psychiatry* 13(924795), 1–8. 10.3389/fpsyt.2022.924795 PMC9709293 36465291

[CIT0020] Liang, Y., Zhou, Y., Ruzek, J.I. & Liu, Z., 2020, ‘Patterns of childhood trauma and psychopathology among Chinese rural to urban migrants’ children’, *Child Abuse and Neglect* 108, 10469. 10.1016/j.chiabu.2020.104691 32854057

[CIT0021] Maepa, M.P. & Ntshalintshali, T., 2020, ‘Family structure and history of childhood trauma: Associations with risk taking behaviour amongst adolescents in Swaziland’, *Frontiers in Public Health* 8(563325), 1–7. 10.3389/fpubh.2020.563325 33154959 PMC7591591

[CIT0022] Mall, S., Platt, J.M., Temmingh, H., Musenge, E., Campbell, M., Susser, E. et al., 2020, ‘The relationship between childhood trauma and schizophrenia in the Genomics of Schizophrenia in the Xhosa people (SAX) study in South Africa’, *Psychological Medicine* 50(9), 1570–1577. 10.1017/S0033291719001703 31387660 PMC7053504

[CIT0023] Manyema, M. & Richter, L.M., 2019, ‘Adverse childhood experiences: Prevalence and associated factors among South African young adults’, *Heliyon* 5(12), 1–10. 10.1016/j.heliyon.2019.e03003 PMC6926197 31890957

[CIT0024] Maroufizadeh, S., Omani-Samani, R., Almasi-Hashiani, A., Amini, P. & Sepidarkish, M., 2019, ‘The reliability and validity of the Patient Health Questionnaiore-9 (PHQ-9) and PHQ-2 in patients with infertility’, *Reported Health* 16(1), 1–9. 10.1186/s12978-019-0802-x PMC6734346 31500644

[CIT0025] Mathijssen, J.J.P., Dirks, E. & Van Bakel, H.J.A., 2024, ‘Transitioning to motherhood: Adverse childhood experiences and support from partner family and friends’, *Maternal and Child Health Journal* 28, 1242–1249. 10.1007/s10995-024-03922-6 38506959 PMC11180152

[CIT0026] McLaughlin, K.A., Colich, N.L., Rodman, A.M. & Weissman, D.G., 2020, ‘Mechanisms linking childhood trauma exposure and psychopathology: A transdiagnostic model of risk and resilience’, *BioMed Central Medicine* 18(96), 1–11. 10.1186/s12916-020-01561-6 PMC7110745 32238167

[CIT0027] Meulewaeter, F., De Pauw, S.S.W. & Vanderplasschen, W., 2019, ‘Mothering, substance use disorders and intergenerational trauma transmission: An attachment-based perspective’, *Frontiers in Psychiatry* 10(728), 1–17. 10.3389/fpsyt.2019.00728 31681040 PMC6813727

[CIT0028] Nowicki, G.J., Schneider-Matyka, D., Godlewska, I., Tytuka, A., Kotus, M., Walec, M. et al., 2023, ‘The relationship between the strength of religious faith and spirituality in relation to post-traumatic growth among nurses caring for COVID-19 patients in eastern Poland: A cross sectional study’, *Frontiers in Psychiatry* 14(1331033), 1–10. 10.3389/fpsyt.2023.1331033 PMC10800582 38260777

[CIT0029] Pennebaker, J.W. & Susman, J.R., 1988, ‘Disclosure of traumas and psychosomatic processes’, *Social Science and Medicine* 26(3), 327–332. 10.1016/0277-9536988)90397-8 3279521

[CIT0030] Petersen, J., Schulz, A.C., Brahler, E., Sachser, C., Fegert, J.M. & Beutel, M.E., 2022, ‘Childhood maltreatment depression and their link to adult economic burdens’, *Frontiers in Psychiatry* 13(908422), 1–13. 10.3389/fpsyt.2022.908422 PMC9441673 36072464

[CIT0031] Rogers, O.J., Foster, M., Sussman, S., Steinberg, J., Barrington-Trimis, J.L., Grigsby, T.J. et al., 2023, ‘The impact of childhood trauma on problematic alcohol and drug use trajectories and moderating role of social support’, *International Journal of Environmental Research and Public Health* 20(2829), 1–13. 10.3390/ijerph20042829 PMC9957226 36833526

[CIT0032] Sharp, T.H., Chideya, Y., Giuliani, A., Hunt, X., Tomlinson, M., Seedat, S. et al., 2024, ‘Post-traumatic stress disorder symptoms following exposure to acute psychological trauma in children aged 8–16 years in South Africa: Protocol for the Sinethemba longitudinal study’, *British Medical Journal* 14, 1–9. 10.31234/osf.io/bqxe8 PMC11624694 38991675

[CIT0033] Singh, M., 2024, ‘Huge investments, poor outcomes, the impact of violence and trauma on learning’, *South African Journal of Childhood Education* 14(1), 1–12. 10.4102/sajce.v14i1.1522

[CIT0034] Spies, G., Kidd, M. & Seedat, S., 2019, ‘A factor analytic study of the childhood trauma questionnaire-short form in all female South African sample with and without HIV’, *Child Abuse and Neglect* 92, 157–166. 10.1016/j.chiabu.2019.04.002 30981158 PMC8853848

[CIT0035] Stansfeld, S.A., Rothon, C., Das-Munshi, J., Matthews, C., Adams, A., Clark, C. et al., 2017, ‘Exposure to violence and mental health of adolescents: South African health and well-being study’, *British Journal of Psychiatry Open* 3(5), 257–264. 10.1192/bjpo.bp.117.004861 29093828 PMC5643877

[CIT0036] StataCorp, 2015, *Stata statistical software: Release 14*, StataCorp LP, College Station, TX.

[CIT0037] Svecova, J., Furstova, J., Kascakova, N., Hasto, J. & Travel, P., 2023, ‘The effects of childhood trauma and resilience on psychopathology in adulthood: Does bullying moderate the association’, *BioMed Central Psychology* 11(230), 1–12. 10.1186/s40359-023-01270-8 PMC10422767 37568213

[CIT0038] Swedo, E.A., Aslam, M.V., Dahlberg, L.L., Niolon, P.H., Guinn, A.S., Simon, T.R. et al., 2023, ‘Prevalence of adverse childhood experiences among US adults-behavioural risk factor surveillance system, 2011–2020’, *Morbidity and Mortality Weekly Report (MMWR)* 72(26), 707–715. 10.15585/mmwr.mm7226a2 37384554 PMC10328489

[CIT0039] Tatala, M., Wojtasinski, M. & Rynio, A., 2024, ‘Religious feelings in early and late adulthood’, *Journal of Beliefs & Values* 47(1), 1–20. 10.1080/13617672.2024.2361496

[CIT0040] Tong, J., Zhang, T., Chen, F., Wang, Q., Zhao, X. & Manji, H., 2022, ‘Prevalence and contributing factors of childhood trauma, anxiety and depression among adolescents from two-child families in China’, *Frontiers in Psychiatry* 13(782087), 1–9. 10.3389/fpsyt.2022.782087 PMC8971896 35370843

[CIT0041] Upenieks, L., Kent, B.V., Nagaswami, M., Gu, Y., Kanaya, A.M. & Shields, A.E., 2024, ‘Do religion and spiritual buffer the effect of childhood trauma on depressive symptoms? Examination of a South Asian Cohort from the USA’, *Journal of Religion and Health* 63(4), 2998–3026. 10.1007/s10943-024-02040-5 38600425 PMC11708237

[CIT0042] Van Zyl, L., Nel, C., Du Toit, M. & Joubert, G., 2017, ‘Reported exposure to trauma among adult patients referred for psychological services at the Free State Psychiatric Complex, Bloemfontein’, *Health SA Gesonheid* 22, 235–240. 10.1016/j.hsag.2017.01.001

[CIT0043] Verma, S. & Agrawal, R., 2021, ‘The lasting effects of childhood trauma’, *Current Psychiatry* 20(3), 18–28. 10.12788/cp.0101

[CIT0044] Wang, S.K., Feng, M., Fang, Y., Liang, L., Sun, G.L., Yang, S.L. et al., 2023, ‘Psychological trauma, post-traumatic stress disorder and trauma-related depression: A mini review’, *World Journal of Psychiatry* 13(6), 331–339. 10.5498/wjp.v13.i6.331 37383283 PMC10294137

[CIT0045] Wang, X., Ding, F., Cheng, C., He, J., Wang, X. & Yao, S., 2022, ‘Psychometric properties and measurement invariance of the childhood trauma questionnaire (short form) across genders, time points and presence of major depressive disorder among Chinese adolescents’, *Frontiers in Psychology* 13(816051), 1–14. 10.3389/fpsyg.2022.816051 PMC9036057 35478747

[CIT0046] Wolkinson, J.T. & Weinberg, M., 2023, ‘Coping with trauma: The relationship between religiosity, spirituality and post-traumatic symptoms among civilians exposed to ongoing rocket fire’, *International Journal of Mental Health Promotion* 25(10), 1137–1145. 10.32604/ijmhp.2023.029641

[CIT0047] Wyatt, G.E., Thames, A., Simbay, L., Stein, D.J., Burns, J. & Maselesele, M., 2017, ‘Trauma and mental health in South Africa: Overview’, *Psychological Trauma* 9(3), 249–251. 10.1037/tra0000144 28459266 PMC5492952

